# High-protein paternal diet confers an advantage to sons in sperm competition

**DOI:** 10.1098/rsbl.2016.0914

**Published:** 2017-02

**Authors:** Felix Zajitschek, Susanne Zajitschek, Mollie Manier

**Affiliations:** 1 Department of Biological Sciences, George Washington University, Washington, DC, USA; 2 School of Biological Sciences, Monash University, Melbourne, Australia; 3 Doñana Biological Station, EBD-CSIC, Seville, Spain

**Keywords:** transgenerational effects, gene expression, parental effects, postcopulatory sexual selection, RNAseq, transcriptomics

## Abstract

Parental environment can widely influence offspring phenotype, but paternal effects in the absence of parental care remain poorly understood. We asked if protein content in the larval diet of fathers affected paternity success and gene expression in their sons. We found that males reared on high-protein diet had sons that fared better during sperm competition, suggesting that postcopulatory sexual selection is subject to transgenerational paternal effects. Moreover, immune response genes were downregulated in sons of low-protein fathers, while genes involved in metabolic and reproductive processes were upregulated.

## 1. Introduction

Parental effects can be triggered by diverse factors and describe non-genetic contributions of parents to offspring developmental phenotypes. Maternal effects are well documented, but less-understood paternal effects can also significantly impact offspring phenotypes [1,2], including sexually selected traits [3–5], even when males contribute only sperm [1,5,6]. Paternal diet, in particular, can influence offspring traits, if females choosing sperm from males adapted to the local nutritional environment produce offspring with higher fitness [7]. Molecular mechanisms of transgenerational paternal diet effects remain poorly understood but include altered methylation in metabolism-linked loci (reviewed in [8]), perturbed glucose–insulin homeostasis [9], altered cholesterol biosynthesis [10], and modified chromatin states related to obesity [6]. Here, we examine how high- and low-protein paternal larval diet influences postcopulatory sexual selection and gene expression in sons of *Drosophila melanogaster.*

## 2. Material and methods

Experimental *D. melanogaster* expressed green fluorescent protein (GFP) in sperm heads and ubiquitously in somatic cells for paternity assignment (focal males) or red fluorescent protein (RFP; females and competitor males) in sperm heads [11]. GFP larvae were reared on high- (HP; 200 g yeast : 50 g sugar) or low-protein (LP; 50 g yeast : 50 g sugar) diet known to yield 80–96% survival [12]. For each treatment, 10 vials were prepared upon eclosion, each with five CO_2_-collected males and five same-stock females reared on standard diet (SD; 100 g yeast : 50 g sugar), housed in SD vials (see electronic supplementary material for more detailed methods). Virgin focal sons were transferred to SD until mating. Three-day-old virgin SD RFP females were first mated with SD RFP competitor males (day 0) in individual vials and provided 6 h opportunities to remate with a focal son for 4 subsequent days (days 1–4) under continuous observation. After remating, females oviposited on fresh SD food vials for 4 days. Paternity of adult offspring [13] was determined using a Nikon SMZ18 fluorescent stereoscope. *P*_2_ was calculated as the proportion of GFP-sired progeny, and data were analysed with logistic regressions with binomial error structure (glm in R v. 3.2.0 [14]).

RNA was extracted from two replicates of 20 7-day-old focal sons per treatment using an RNeasy kit (Qiagen) and quantified using Agilent Bioanalyzer. Illumina TruSeq mRNA stranded libraries were constructed, and 76 bp paired-end sequences were obtained on an Illumina NextSeq 500, replicated across two flow cells, with within-sample replicates pooled for further analysis [15]*.* We performed RNASeq data analysis using the Tuxedo Protocol in the DNA Subway online platform [16] with quality control using FastX-Toolkit (v. 0.0.13.2). Reads were mapped to the *D. melanogaster* transcriptome and genome (Ensembl r76, BDPG5) using TopHat (v. 2.0.11, [17]). Differentially expressed (DE) genes were identified using CUFFDIFF (v. 2.1.1, [16]) at a *q*-value < 0.05 after false discovery rate correction [18]. Results were visualized with CummeRbund and Cytoscape (for biological networks, [19]) in R.

## 3. Results

**Figure 1.**
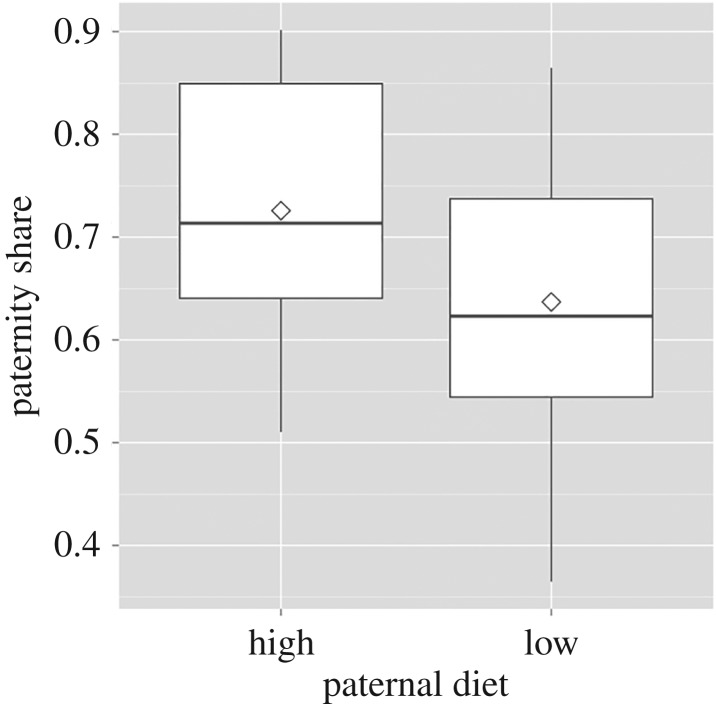
Paternity share (*P*_2_) of sons from fathers on either high or low larval diet.

**Figure 2.**
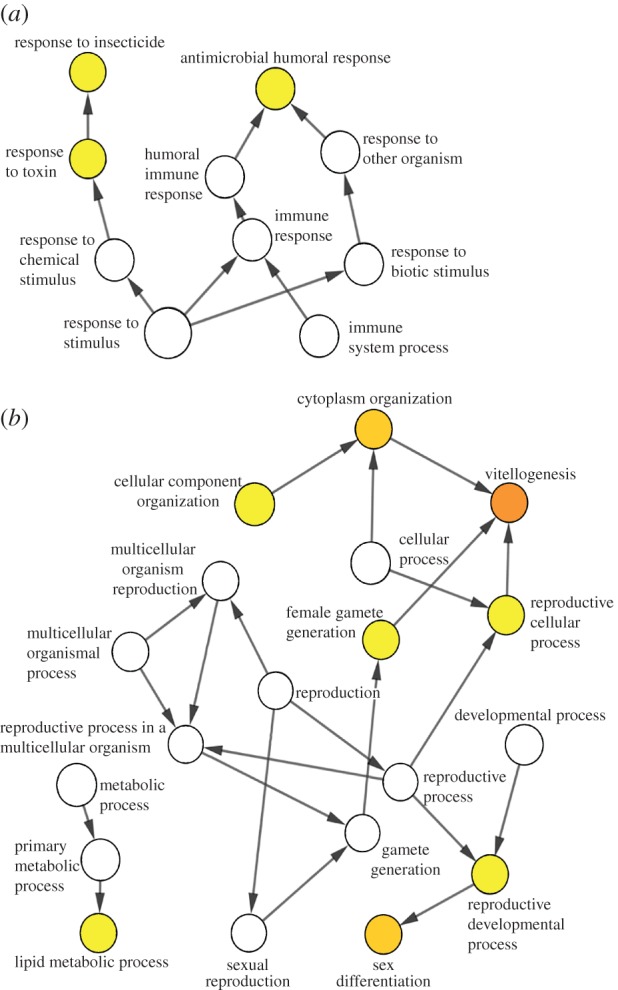
(*a*) Downregulated gene clusters with GO (gene ontology) terms in sons of fathers on low diet. (*b*) Upregulated gene clusters with GO terms in sons of fathers on low diet. Nodes with significantly enriched GO terms are shown in colour.

*P*_2_ of sons from fathers on high larval diet was higher than that of sons from low larval diet fathers (estimate ± s.e. = −0.216 ± 0.077, *Z* = –2.80, *p* = 0.005; figure 1). Of 69 DE genes (*q* ≤ 0.05; fold change > 1.5), 58 were downregulated (fold change 1.54–10.6; mean ± s.e. = 2.30 ± 1.46) in LP sons related to immune response, specifically antimicrobial humoral response and response to insecticides and other toxins (figure 2*a*). Eleven genes were upregulated primarily in reproductive and metabolic functions (fold change 1.66–6.2; mean ± s.e. = 2.83 ± 1.54; see table 1, electronic supplementary material table S1 and figure 2*b*).

**Table 1.** Differentially expressed genes in sons (*q* ≤ 0.05) at ≥ 2-fold. FPKM, fragments per kilobase of transcript per million reads mapped. If no further information on a gene is available, cells have been left blank.

gene	fold change	direction (low diet)	high diet (FPKM)	low diet (FPMK)	*q*-value	description (gene product)	biological function
AttD	10.6	down	62.98	5.94	0.0171	attacin-D	antimicrobial
CG8534	6.45	down	3.34	0.52	0.0171		fatty acid elongation
para	6.31	down	1.54	0.24	0.0171	paralytic	courtship song
Yp1	6.2	up	0.73	4.55	0.0171	yolk protein 1	seminal vesicle protein
Yp2	5.49	up	0.88	4.84	0.0171	yolk protein 2	seminal vesicle protein
CG11873	5	down	3.31	0.66	0.0171		response to endoplasmic reticulum stress
CG42795	3.62	down	1.97	0.55	0.0171		regulation of GTPase activity
Cpr92F	2.8	down	3.47	1.24	0.0171	cuticular protein 92F	chitin-based cuticle development
Jeb	2.73	down	1.84	0.67	0.0171	jelly belly	various
CG9377	2.67	up	3.03	8.09	0.0171		proteolysis
Dp	2.62	down	1.77	0.68	0.0171	dumpy	chitin-based embryonic cuticle biosynthetic process
CG40472	2.55	up	10.91	27.87	0.0171		mitochondrial respiratory chain complex I
mei-P26	2.47	down	1.75	0.71	0.0171	mei-P26	gamete generation
DopR	2.31	down	0.62	0.27	0.0171	dopamine receptor	learning
Ace	2.29	down	11.17	4.87	0.0171	acetylcholine esterase	catabolic process
zfh2	2.27	down	2.02	0.89	0.0171	Zn finger homeodomain 2	nervous system development
Ca-alpha1T	2.16	down	2.26	1.04	0.03	Ca^2+^-channel protein alpha 1 subunit T	calcium ion import
CG30069	2.13	down	6.38	3.00	0.0171		
CR40685	2.13	down	6.81	3.20	0.03		
Scrt	2.11	down	2.46	1.16	0.0171	scratch	dendrite morphogenesis
CR40469	2.11	up	274.25	577.54	0.0171		
Corin	2.1	down	1.23	0.59	0.0171	Corin	proteolysis
Dp	2.1	down	1.25	0.59	0.0171	dumpy	
Ac3	2.09	down	1.58	0.75	0.0171	Ac3	cAMP biosynthetic process
CG13185	2.08	down	1.71	0.82	0.0171		cellular response to starvation
Kst	2.02	down	19.76	9.78	0.0171	karst	microtubule binding
Yp3	2	up	4.68	9.38	0.0171	yolk protein 3	neurogenesis

## 4. Discussion

Sons of fathers reared on LP diet fared worse in sperm competition, with associated downregulation of immune response genes and upregulation of genes involved in metabolism and reproduction. Non-mutually exclusive mechanisms of paternal effects on paternity success include seminal fluid and other ejaculate effects [20] and cryptic female choice [21]. Females may have been able to detect treatment-induced variation in male behaviour and may have allocated more resources into reproduction with descendants of high-diet males. It is well known that high-quality diet positively affects male sexual characters [22], fitness [23] and subsequent female choice [24]. Indeed, the gene *paralytic* (*para*) affects courtship song [25] and male olfaction in response to female pheromones [26] and was downregulated in sons of LP fathers. As downregulation of *para* reduces neuronal excitability [27], it is conceivable that negative fitness effects include lower-quality courtship song and reduced olfaction ability, which are very important factors in female precopulatory choice [28]. However, while higher latency (willingness) to mate and reduced mating duration for males with low-quality courtship song and reduced olfactory ability may be expected, we did not find an effect of paternal diet regime on mating duration, and we did not investigate more detailed behavioural traits to confirm correlational outcomes with the expression of *para*. Only few studies have so far reported transgenerational effects in relation to diet quality [29,30]. To our knowledge, this is the first study reporting on postcopulatory advantages conferred by parental diet.

Importantly, DE genes confirm the existence of differences between sons of fathers reared on different diets, enabling further investigations of transgenerationally affected sexually selected traits. Antimicrobial peptides (AMPs) are upregulated by *D. melanogaster* when challenged by Gram-negative bacteria [31,32]. Downregulation of these AMPs in sons of LP fathers in our study might therefore be a form of immunosuppression, which, according to theory, trades off against sexually selected traits [33]. Thus, reproductive fitness of LP sons might have been even lower if immunosuppression had not occurred. Indeed, sexually selected male *D. melanogaster* that showed higher competitive mating ability had lowered immune function, compared with control males [34].

The two most upregulated genes in sons of low-diet fathers are *YP1* and *YP2*. While the suggested functional annotation, vitellogenesis, is clearly a female-limited function, effects of *YP1* and *YP2* in male *D. melanogaster* [35] and the moth *Spodoptora littoralis* [36] include yolk protein precursors, which directly interact with spermatozoa. *YP2* coats the spermatozoa and might provide protection or aid in gamete recognition. However, the functional significance of these proteins has not been established, and we have no knowledge about how upregulation of *YP1* and *YP2* may influence reproductive fitness in male fruit flies.

The direction of regulation of proteolysis (*CG9377*), biosynthesis of chitin-based cuticle (*Cpr92F* and *dp*) and gamete generation (*mei-P26*) is consistent with organismal preparation for a suboptimal nutritional environment, investing less and recycling more. Intriguingly, *CG9377* has been also found to be upregulated in brains of male *D. melanogaster* courting females, compared with non-courting males [37], establishing another link of our paternal diet treatment to precopulatory sexual selection (although the direction of the effect seems to promote courtship, rather than reduce it, as discussed above). Valtonen *et al*. [38] found substantial transgenerational effects of larval diet on development time and adult body size in *D. melanogaster*, but not on pathogen resistance. The different findings in immune response between [38] and the presented study may be due to the efficiency of the manipulated media. Diet components and protein : carbohydrate ratios are difficult to compare between studies, owing to use of different protein (P) and carbohydrate (C) sources. Crude estimates of P : C ratios and the within-study difference between ratios were much higher in our study (low = 0.4, high = 8; [38] assuming 100 g of sugar/litre diet: low = 0.07, standard = 0.14), illustrating the need to employ a more exact nutritional framework to determine high-resolution reaction norms of traits of interest [39].

## Supplementary Material

Supplemental material

## Data accessibility

Data on paternity success are archived in Dryad (http://dx.doi.org/10.5061/dryad.9qs53) [13]; sequencing reads are deposited in NCBI under BioProject number PRJNA360276.
