# Supplementary material for: High-protein paternal diet confers an advantage to sons in sperm competition
Source: Biol Lett. 2017 Feb;13(2):20160914. doi: 10.1098/rsbl.2016.0914 (PMC5326516; doi:10.1098/rsbl.2016.0914)
Supplement: Supplemental material [file rsbl20160914supp1.docx]

High protein paternal diet confers an advantage to sons in sperm competition

Felix Zajitschek^1,2^ *, Susanne Zajitschek^1,2,3^ * and Mollie Manier^1^

* joint first authors

^1^ Department of Biological Sciences, George Washington University, Washington, DC, USA

^2^ School of Biological Sciences, Monash University, Melbourne, Australia

^3^ Doñana Biological Station, EBD-CSIC, Seville, Spain

***Supplementary Methods***

***Supplementary Table S1***

**Methods**

**Diet manipulation and paternity success**

We used transgenic LHm D. melanogaster expressing green (GFP) or red fluorescent protein (RFP) in sperm heads. Details on transformations can be found in Manier et al. (2010). GFP flies also express GFP ubiquitously, allowing paternity assignment after sperm competition with RFP. We exposed larvae to two diet treatments, differing in their yeast:sugar ratios, with 50g yeast:50g sugar as a low protein treatment and 200g yeast:50g sugar as high protein treatment (following Bass et al., 2007). Larval diets with yeast:sugar ratios in this range are based on the macronutrient variation in natural host choices in this species (Markow et al., 1999) and result in 80 to 96% survival to adulthood (Matzkin et al., 2011). Upon eclosion, male flies were transferred to standard diet (SD; 100g yeast : 50g sugar) and mated 7 days later to same-age, SD-reared virgin female GFP flies to generate the experimental focal sons. Females were allowed to oviposit on SD, and the resulting focal sons remained on SD throughout the experiment, and all other fly-rearing and matings occurred on SD. All larval densities were low (< 100 larvae per vial) to minimize competition, and flies were reared in constant-temperature chambers at 25°C under a 12:12 light-dark cycle.

To assess sperm competitiveness of focal GFP sons, RFP females were first mated with RFP competitor males at age 3 days, and provided a 4-hour opportunity to remate with focal GFP sons from either paternal diet treatment for 4 consecutive days after remating. Pairs were continuously observed for successful mating for 6 hours/day. After mating, females were transferred to a fresh food vial allowed to oviposit for four days. Paternity was scored on adult offspring under fluorescent light, using the presence/absence of ubiquitous GFP expression as a paternal marker. Due to low success rates for double matings (30 %) we obtained paternity data for only 42 males (N_low_ = 28, N_high_ = 16). The paternity data were analysed with logistic regressions with binomial error structure (glm in R 3.2.0).

**Differential gene expression**

RNA extraction

We followed the same protocol to obtain focal males as described above. Two technical replicates of 20 sons per treatment were snap-frozen in liquid nitrogen and stored in 800 µl RNAlater at 7 days of adult age. Total RNA was extracted using the miRNeasy 96 kit (Qiagen), according to the manufacturer’s protocol. Total amount of RNA was quantified using a Nanodrop X spectrophotometer (Thermo Scientific; low diet treatment, sample 1: 60 µl with 92 ng/µl, sample 2: 62 µl with 112 ng/µl; high diet treatment, sample 1: 45 µl with 75 ng/µl, sample 2: 45 µl with 57 ng/µl).

RNA-Seq for differential gene expression

Samples were converted into an Illumina TruSeq mRNA stranded library and sequenced by 76-bp paired ends in two flow cells on an Illumina NextSeq 500 sequencer. Flow cell replicates within samples were pooled for further analyses. We performed data analysis using the Tuxedo Protocol (Trapnell et al., 2012) in the DNA Subway online platform ([Green Line pipeline under www.dnasubway.org, Hilgert et al. 2014](#_ENREF_7)). Quality control was performed using the FASTX-Toolkit (v0.0.13.2). After quality control, approximately 20 million reads per sample were analysed. Reads were mapped to the D. melanogaster transcriptome and genome (Ensembl r76, BDPG5) using TopHat (v2.0.11, Trapnell et al., 2012). Expression levels and differential expression were analysed using CUFFDIFF ([v2.1.1, Trapnell et al. 2012](#_ENREF_17)), with genes assumed to be differentially expressed given a q value less than 0.05, after Benjamini and Hochberg false discovery rate correction (Benjamini and Hochberg, 1995). Results were visualized with the R package CummeRbund, and with Cytoscape (for biological networks, Smoot et al., 2011).

References

*1. Bass TM, Weinkove D, Houthoofd K, Gems D, Partridge L. Effects of resveratrol on lifespan in Drosophila melanogaster and Caenorhabditis elegans. Mech Ageing Dev. 2007 Oct;128(10):546–52.*

*2. Matzkin LM, Johnson S, Paight C, Bozinovic G, Markow TA. Dietary protein and sugar differentially affect development and metabolic pools in ecologically diverse Drosophila. J Nutr. 2011 Jun;141(6):1127–33.*

*3. Markow TA, Raphael B, Breitmeyer CM, Dobberfuhl D, Elser J, Pfeiler EJ. Elemental stoichiometry of Drosophila and their hosts. Funct Ecol. 1999;13(1):78–84.*

*4. Trapnell C, Roberts A, Goff L, Pertea G, Kim D, Kelley DR, et al. Differential gene and transcript expression analysis of RNA-seq experiments with TopHat and Cufflinks. Nat Protoc. 2012 Mar;7(3):562–78.*

*5. Benjamini Y, Hochberg Y. Controllong the false discovery rate - a practical and powerful approach to multiple testing. J R Stat Soc Ser B-Methodol. 1995;57(1):289–300.*

*6. Smoot ME, Ono K, Ruscheinski J, Wang P-L, Ideker T. Cytoscape 2.8: new features for data integration and network visualization. Bioinforma Oxf Engl. 2011 Feb 1;27(3):431–2.*

Table S1. Differentially expressed genes in sons with Q-value ≤ 0.05, and a fold-change of more than 1.5. FPKM: fragments per kilobase of transcript per million reads mapped. If no further information on a gene is available, cells have been left blank.

| gene | | **fold change** | | **direction (low diet)** | **high diet (FPKM)** | **low diet (FPMK)** | **Q-Value** | **description (gene product)** | **biological function** |
| --- | --- | --- | --- | --- | --- | --- | --- | --- | --- |
| AttD | 10.6 | down | | 62.98 | 5.94 | 0.0171 | Attacin-D | antimicrobial |  |
| CG8534 | 6.45 | down | | 3.34 | 0.52 | 0.0171 |  | fatty acid elongation |  |
| para | 6.31 | down | | 1.54 | 0.24 | 0.0171 | paralytic | courthship song |  |
| Yp1 | 6.2 | up | | 0.73 | 4.55 | 0.0171 | Yolk protein 1 | seminal vesicle protein |  |
| Yp2 | 5.49 | up | | 0.88 | 4.84 | 0.0171 | Yolk protein 2 | seminal vesicle protein |  |
| CG11873 | 5 | down | | 3.31 | 0.66 | 0.0171 |  | response to endoplasmic reticulum stress |  |
| CG42795 | 3.62 | down | | 1.97 | 0.55 | 0.0171 |  | regulation of GTPase activity |  |
| Cpr92F | 2.8 | down | | 3.47 | 1.24 | 0.0171 | Cuticular protein 92F | chitin-based cuticle development |  |
| jeb | 2.73 | down | | 1.84 | 0.67 | 0.0171 | jelly belly | various |  |
| CG9377 | 2.67 | up | | 3.03 | 8.09 | 0.0171 |  | proteolysis |  |
| dp | 2.62 | down | | 1.77 | 0.68 | 0.0171 | dumpy | chitin-based embryonic cuticle biosynthetic process |  |
| CG40472 | 2.55 | up | | 10.91 | 27.87 | 0.0171 |  | mitochondrial respiratory chain complex I |  |
| mei-P26 | 2.47 | down | | 1.75 | 0.71 | 0.0171 | mei-P26 | gamete generation |  |
| DopR | 2.31 | down | | 0.62 | 0.27 | 0.0171 | Dopamine receptor | learning |  |
| Ace | 2.29 | down | | 11.17 | 4.87 | 0.0171 | Acetylcholine esterase | catabolic process |  |
| zfh2 | 2.27 | down | | 2.02 | 0.89 | 0.0171 | Zn finger homeodomain 2 | nervous system development |  |
| Ca-alpha1T | 2.16 | down | | 2.26 | 1.04 | 0.03 | Ca[2+]-channel protein alpha[[1]] subunit T | calcium ion import |  |
| CG30069 | 2.13 | down | | 6.38 | 3.00 | 0.0171 |  |  |  |
| CR40685 | 2.13 | down | | 6.81 | 3.20 | 0.03 |  |  |  |
| scrt | 2.11 | down | | 2.46 | 1.16 | 0.0171 | scratch | dendrite morphogenesis |  |
| CR40469 | 2.11 | up | | 274.25 | 577.54 | 0.0171 |  |  |  |
| Corin | 2.1 | down | | 1.23 | 0.59 | 0.0171 | Corin | proteolysis |  |
| dp | 2.1 | down | | 1.25 | 0.59 | 0.0171 | dumpy |  |  |
| Ac3 | 2.09 | down | | 1.58 | 0.75 | 0.0171 | Ac3 | cAMP biosynthetic process |  |
| CG13185 | 2.08 | down | | 1.71 | 0.82 | 0.0171 |  | cellular response to starvation |  |
| kst | 2.02 | down | | 19.76 | 9.78 | 0.0171 | karst | microtubule binding |  |
| Yp3 | 2 | up | | 4.68 | 9.38 | 0.0171 | Yolk protein 3 | neurogenesis |  |
| CG4525 | 1.99 | up | | 1.69 | 3.37 | 0.0171 |  | cilium assembly |  |
| Smr | 1.98 | down | | 3.69 | 1.86 | 0.0171 | Smrter | chromatin binding |  |
| bru-3 | 1.95 | down | | 2.67 | 1.37 | 0.0171 | bruno-3 | RNA binding |  |
| Rdl | 1.95 | down | | 3.49 | 1.79 | 0.0171 | Resistant to dieldrin | GABA-A receptor activity |  |
| Dpt | 1.91 | down | | 879.57 | 459.89 | 0.0171 | Diptericin | antibacterial humoral response |  |
| bt | 1.91 | down | | 33.33 | 17.48 | 0.0171 | bent |  |  |
| CG10778 | 1.91 | up | | 6.04 | 11.53 | 0.03 |  |  |  |
| ptip | 1.9 | down | | 2.30 | 1.21 | 0.0171 |  |  |  |
| CG33988 | 1.89 | down | | 2.31 | 1.22 | 0.0171 |  |  |  |
| Rbp6 | 1.88 | down | | 5.71 | 3.04 | 0.0171 | RNA-binding protein 6 |  |  |
| CG15358 | 1.86 | down | | 15.14 | 8.15 | 0.0171 |  |  |  |
| kl-2 | 1.85 | down | | 0.71 | 0.39 | 0.03 | male fertility factor kl2 | microtubule-based movement |  |
| Ncoa6 | 1.85 | down | | 5.61 | 3.03 | 0.0171 |  |  |  |
| SK | 1.84 | down | | 2.72 | 1.48 | 0.0171 | small conductance calcium-activated potassium channel |  |  |
| dysc | 1.84 | down | | 2.49 | 1.35 | 0.03 | dyschronic |  |  |
| cac | 1.81 | down | | 2.72 | 1.50 | 0.0171 | cacophony | courtship song |  |
| CG43901 | 1.81 | down | | 1.35 | 0.75 | 0.0171 |  |  |  |
| CG42450 | 1.8 | down | | 4.00 | 2.22 | 0.0171 |  |  |  |
| Eip93F | 1.78 | down | | 12.49 | 7.00 | 0.0171 | Ecdysone-induced protein 93F |  |  |
| CG34113 | 1.78 | down | | 1.30 | 0.73 | 0.0437 |  |  |  |
| Kul | 1.77 | down | | 4.95 | 2.79 | 0.0171 | Kuzbanian-like |  |  |
| Muc68E | 1.76 | down | | 3.89 | 2.21 | 0.0171 | Mucin 68E |  |  |
| mt:ND2 | 1.75 | up | | 93.14 | 162.92 | 0.0171 | mitochondrial NADH-ubiquinone oxidoreductase chain 2 |  |  |
| Mur29B | 1.74 | down | | 48.79 | 28.08 | 0.0171 | Mucin related 29B |  |  |
| mgl | 1.74 | down | | 10.62 | 6.10 | 0.0171 | Megalin |  |  |
| Rfabg | 1.72 | down | | 228.54 | 132.80 | 0.0171 | Retinoid- and fatty acid-binding glycoprotein |  |  |
| CG15635 | 1.71 | down | | 16.82 | 9.85 | 0.0171 |  |  |  |
| CG9007 | 1.69 | down | | 8.57 | 5.07 | 0.0171 |  |  |  |
| CG10011 | 1.67 | down | | 4.87 | 2.92 | 0.0171 |  |  |  |
| CG8677 | 1.66 | down | | 6.70 | 4.04 | 0.0171 |  |  |  |
| CG12224 | 1.66 | up | | 170.44 | 283.25 | 0.0171 |  |  |  |
| hyd | 1.62 | down | | 25.88 | 15.96 | 0.0171 | hyperplastic discs |  |  |
| kibra | 1.62 | down | | 6.96 | 4.29 | 0.0171 | kibra ortholog |  |  |
| CG31817 | 1.62 | down | | 11.40 | 7.06 | 0.0171 |  |  |  |
| CG42255 | 1.58 | down | | 2.95 | 1.87 | 0.0171 |  |  |  |
| CG10777 | 1.57 | down | | 14.03 | 8.95 | 0.03 |  |  |  |
| CG8184 | 1.57 | down | | 11.21 | 7.15 | 0.0171 |  |  |  |
| eIF4G2 | 1.57 | down | | 52.63 | 33.42 | 0.0171 | eukaryotic translation initiation factor 4G2 |  |  |
| CG15523 | 1.55 | down | | 23.31 | 14.99 | 0.03 |  |  |  |
| Dhc98D | 1.55 | down | | 3.81 | 2.46 | 0.03 | Dynein heavy chain at 89D |  |  |
| CG3523 | 1.54 | down | | 179.87 | 116.68 | 0.0437 |  |  |  |
